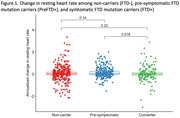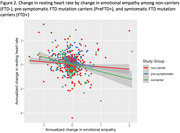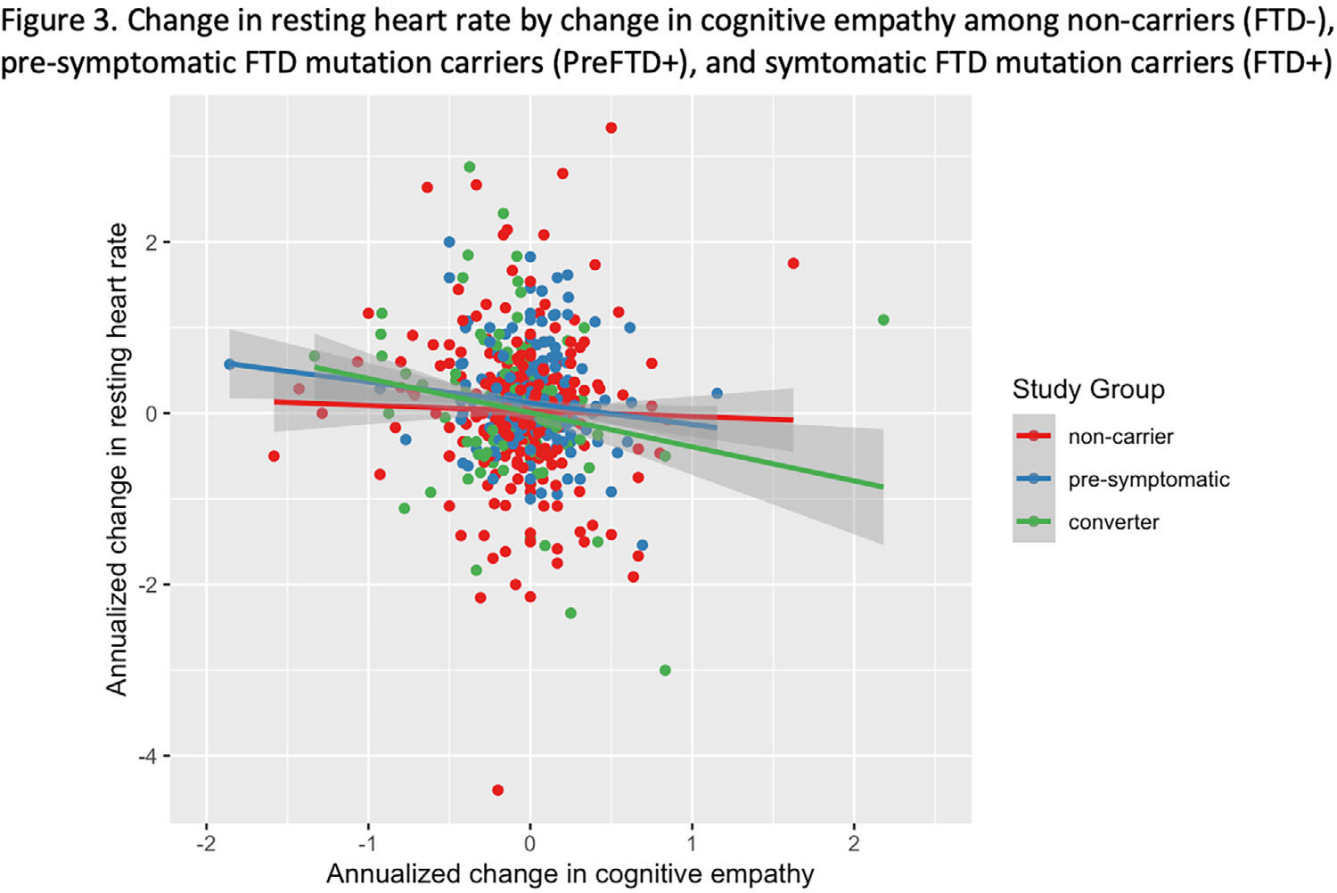# Longitudinal variability in resting heart rate associates with empathy decline in pre‐symptomatic and symptomatic familial frontotemporal degeneration

**DOI:** 10.1002/alz.091893

**Published:** 2025-01-03

**Authors:** Emma Rhodes, Lauren Massimo, Laynie Dratch, David J Irwin, Brad F. Boeve, Hilary W. Heuer, Adam L. Boxer, Howard J. Rosen, Katherine P. Rankin, Virginia Sturm, Corey T McMillan, ARTFL‐LEFFTDS (ALLFTD)

**Affiliations:** ^1^ Frontotemporal Degeneration Center, University of Pennsylvania, Philadelphia, PA USA; ^2^ Penn Frontotemporal Degeneration Center, Department of Neurology, Perelman School of Medicine, University of Pennsylvania, Philadelphia, PA USA; ^3^ Penn Frontotemporal Degeneration Center, University of Pennsylvania, Philadelphia, PA USA; ^4^ Department of Neurology, Mayo Clinic, Rochester, MN USA; ^5^ University of California, San Francisco, San Francisco, CA USA; ^6^ Memory and Aging Center, Weill Institute for Neurosciences, University of California, San Francisco, San Francisco, CA USA; ^7^ University of California San Francisco, San Francisco, CA USA; ^8^ Frontotemporal Degeneration Center, Department of Neurology, Perelman School of Medicine, University of Pennsylvania, Philadelphia, PA USA; ^9^ ARTFL‐LEFFTDS Longitudinal Frontotemporal Lobar Degeneration (ALLFTD) Research Consortium, CA USA

## Abstract

**Background:**

Autonomic dysfunction has been linked to empathy deficits in symptomatic frontotemporal degeneration (FTD), but less is known about pre‐symptomatic FTD mutation carriers (preFTD+). Our prior work found that increasing resting heart rate (RHR) over time predicts decline in emotional empathy in preFTD+. Here, we replicate previous findings in a large, multi‐site consortium sample and assess relationships between RHR and empathy loss across disease stages.

**Method:**

RHR and Interpersonal Reactivity Index (IRI) were obtained serially for up to 7 years in 348 preFTD+, 202 symptomatic FTD mutation carriers (FTD+), and 311 non‐carrier family members (FTD‐) enrolled in ALLFTD. IRI Perspective Taking (IRI‐PT) and Empathic Concern (IRI‐EC) subscales measured cognitive and emotional empathy, respectively. Separate multiple linear regressions related mutation status and annualized change in RHR to annualized change in IRI subscales and assessed interactions between RHR change and phenoconversion status among mutation carriers. Logistic regression was used to assess the association between change in RHR and phenoconversion among mutation carriers, and Wilcoxon signed‐rank tests were used to examine differences in change in RHR between all study groups.

**Result:**

In asymptomatic participants (preFTD+ and FTD‐), increase in RHR over time was associated with greater decline in IRI‐EC (ß = ‐0.07, p<.001) and IRI‐PT (ß = ‐0.03, p = .011), regardless of mutation status. Among mutation carriers (preFTD+ and FTD+), increase in RHR was associated with greater decline in IRI‐EC (ß = ‐0.04, p = .041), and this effect was stronger in FTD+ compared to PreFTD+ (ß = ‐0.07, p = .038), while an increase in RHR was associated with decline in IRI‐PT only in FTD+ (ß = ‐0.07, p = .046). Change in RHR did not predict phenoconversion in mutation carriers (PreFTD+ and FTD+), but PreFTD+ showed less change in RHR over time relative to FTD+.

**Conclusion:**

Changes in empathy in asymptomatic preFTD+ and FTD‐ are linked to variability in RHR regardless of genetic status, likely reflecting a non‐specific impact of autonomic functioning on empathy and prosocial behavior. In contrast, increasing RHR predicts decline in cognitive and emotional empathy in FTD mutation carriers but primarily after phenoconversion, suggesting that autonomic contributions to empathy in familial FTD increase with disease progression and could be an early marker of manifest disease.